# Laser speckle contrast imaging to monitor microcirculation: An effective method to predict outcome in patients with sepsis and septic shock

**DOI:** 10.3389/fbioe.2022.1067739

**Published:** 2023-01-11

**Authors:** Zhengshang Ruan, Ran Li, Wenwen Dong, Zhilei Cui, Hui Yang, Rongrong Ren

**Affiliations:** ^1^ Department of Infectious Diseases, Xinhua Children’s Hospital, Xinhua Hospital, Shanghai Jiao Tong University School of Medicine, Shanghai, China; ^2^ School of Optical-Electrical and Computer Engineering, University of Shanghai for Science and Technology, Shanghai, China; ^3^ Department of Anesthesiology and Surgical Intensive Care Unit, Xinhua Hospital, Shanghai Jiaotong University School of Medicine, Shanghai, China; ^4^ Department of Respiratory Medicine, XinHua Hospital Affiliated to Shanghai Jiao Tong University School of Medicine, Shanghai, China

**Keywords:** septic shock, laser speckle contrast imaging, perfusion index, sepsis, APACH II score

## Abstract

**Background:** This study examines the microcirculation of patients with sepsis and septic shock using Laser Speckle Contrast Imaging (LSCI) technology, to enhance monitoring and predict outcomes of sepsis and septic shock.

**Methods:** From 01 July 2021, to 31 January 2022, 44 patients diagnosed with septic shock and sepsis were included in the study, their clinical data were collected, and LSCI was used to monitor the mean peripheral blood flow perfusion index (PI).

**Results:** The average peripheral blood flow PI of septic shock patients was significantly lower than that of septic patients, with a cutoff value of 26.25. The average peripheral blood flow PI negatively correlated with acute physiology and chronic health evaluation (APACHE) Ⅱ score (*p* = .01 < .05), sequential organ failure assessment (SOFA) score (*p* < .01), and lactic acid levels (*p* = .01 < .05). We report average peripheral blood flow no correlation with age, mean arterial pressure, body temperature, oxygen saturation, heart rate, and body mass index. There was no correlation with procalcitonin, C-reactive protein (CRP), red blood cell distribution width, or platelet distribution width (*p* > .05). PI significantly correlated with the group sepsis and septic shock (*p* < .001, r = −.865). And PI significantly correlated with the outcome or mortality (*p* = .007 < .05, r = −.398). The ROC curve was calculated for PI and the sensitivity was 81.3%, and the specificity was 75% when PI cutoff value chooses 20.88.

**Conclusion:** LSCI technology successfully detected the fingertip microcirculation of patients with septic shock. LSCI can reliably differentiate patients with sepsis vs patients with septic shock. Additionally, the average peripheral blood PI negatively correlated with APACHE Ⅱ, SOFA score, and lactate acid levels, providing useful and supplementary information for the diagnosis and monitoring of septic shock. Trial registration: Chictr2100046761. Registered on May 28, 2021.

**Clinical Trial Registration:** clinicaltrials.gov, identifier Chictr2100046761

## Introduction

Sepsis typically results from infection and, if left unchecked, can lead to septic shock and subsequent potentially life-threatening organ damage. Both conditions are associated with high mortality, ultimately resulting in a heavy burden on the healthcare system (Hotchkiss and Karl, 2003; Cecconi et al., 2018). In 2020, a study report examining 44 intensive care units (ICUs) in China showed that the incidence rate of sepsis was 20.6%. Further analysis of 170 studies found that the average mortality of septic shock patients at 30 and 90 days were 34.7% (95% CI 32.6%–36.9%) and 38.5% (95% CI 35.4%–41.5%), respectively (Emergency Medicine Branch Of Chinese Medical Care International Exchange Promotion Association, Emergency Medical Branch Of Chinese Medical Association, Chinese Medical Doctor Association Emergency Medical Branch, and Chinese People’s Liberation Army Emergency Medicine Professional Committee, 2020). The pathophysiology of septic shock revolves around adequate hemodynamic function. Specifically, reduced circulation leads to impaired microcirculation perfusion and decreased tissue and organ function (Kanoore Edul et al., 2011). Since there is a close and direct correlation between septic shock induced changes in microcirculation and mortality, the recognition and correct evaluation of microcirculation dysfunction in sepsis can guide the clinical treatment of septic shock.

Sepsis is known to induce alterations in microvascular perfusion such as decreased perfusion vessel density (functional capillary density) and perfusion heterogeneity between regions (near several microns). In a study that was subsequently replicated in over 30 papers, De Backer et al. (2002) showed that in patients with sepsis demonstrated sublingual microcirculation alterations through decreased density of perfusion vessels, increased heterogeneity, and the presence of non-perfused capillaries near the perfusion vessels.

As direct examination of internal organs, such as the kidney, liver, heart or brain, is not feasible, most of the current experimental studies focus on the sublingual region, or, recently, on the conjunctival region (Simkiene et al., 2020). The monitoring of sublingual microcirculation relies on side-stream dark field (SDF) imaging technology (Zhao et al., 2020). The disadvantage of this technology is that sublingual saliva bubbles may affect measurement accuracy. Moreover, this method becomes more difficult in patients with endotracheal intubation. Laser speckle contrast imaging (LSCI) (Barcelos et al., 2017) is based on the laser irradiation of red blood cells to generate a speckle image which is then used to obtain blood flow velocity. This is done by calculating the speckle contrast value to generate a full-field two-dimensional and high-resolution image of blood flow distribution. Compared with other methods of clinical monitoring of blood microcirculation, this technology can not only achieve long-term continuous blood flow monitoring, but also requires no contact, is non-invasive, has high temporal and spatial resolution, and uses large-area rapid imaging.

Perfusion assessment using LSCI in rheumatology shows promising results in determining the state of systemic sclerosis (Della Rossa et al., 2013; Rosato et al., 2013) characterize different stages of arthritis (Son et al., 2014) and evaluate the activity of rheumatological diseases (Bray et al., 2006). LSCI is used to assess superficial blood flow within burn surgery and to predict wound healing (Stewart et al., 2005). LSCI is also widely used in ophthalmology, gastroenterology, neurology, dentistry and other fields of medicine, and is currently one of the largest fields of research (Heeman et al., 2019).

This study used LSCI to monitor the microcirculation of the fingertips of patients with sepsis and septic shock, to enhance the monitoring and diagnosis of this condition.

## Materials and methods

### Settings and study population

The research protocol got approval of the hospital ethics committee (approval no. xhec-d-2014-005). All patients and their family members provided informed consent. The clinical trial registration number is: chictr2100046761. Patients over 18 years old, with sepsis and septic shock diagnosis according to sepsis guideline 2016 (Rhodes et al., 2017), obtained at the Department of Anesthesiology and critical care at Xinhua Hospital from 1 July 2021, to 31 January 2022, were included in the study. We excluded patients who declined study participation; Patients with limb fracture, trauma and other reasons that prevent measuring of the microcirculation; patients with diabetes, upper limb thromboembolism; patients required to use an ice blanket or had any other diseases affecting skin blood circulation or serious organic diseases; pregnant women. Patient data were collected within 1 h after admission to the ICU or within 1 hour after being diagnosed with sepsis or septic shock.

### Laser speckle contrast imaging

**FIGURE 1:**
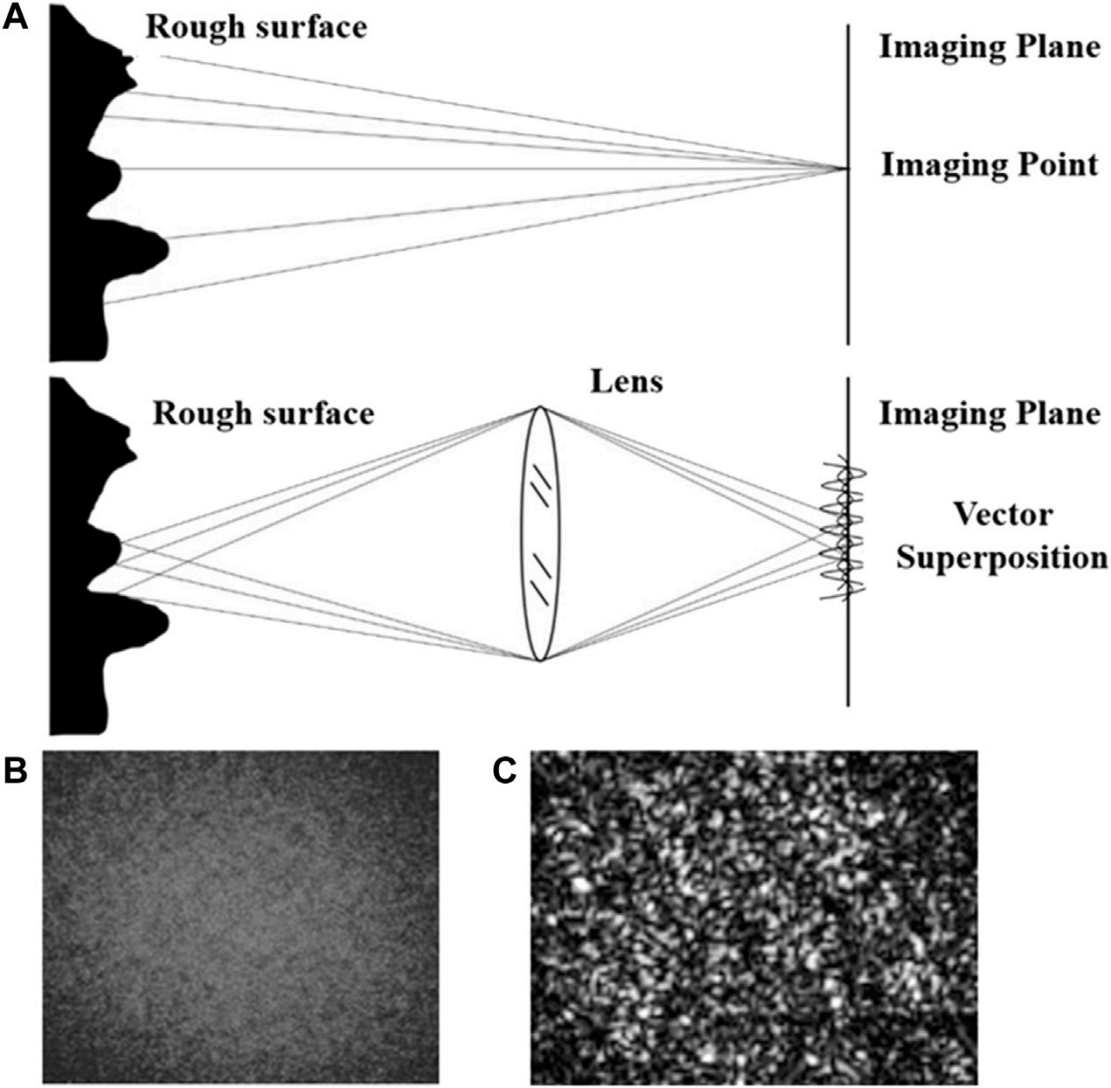
Principle of speckle imaging **(A)** Theoretical schematic diagram of the speckle imaging; **(B)** Original speckle imaging; **(C)** Speckle imaging in grayscale, range [0, 255].

Laser speckle tracking uses irradiation to create a spatial fluctuation of light on the surface of an object (Dunn et al., 2001; Voigt et al., 2015). These light and dark alternating and randomly arranged luminous particles are speckle patterns. Speckle data analysis can be divided into speckle spectrum and speckle imaging methods, as shown in Figure 1A, which can be distinguished according to whether there is a lens between the imaging object and the imaging surface.

The patterns formed by superimposing diffuse light refracted back from the object surface collected with a lens are called subjective speckle or imaging speckle, as shown in Figure 1B. Due to the existence of the object-image relationship, the phase amplitude vector of a point on the receiving plane is the vector superposition of the phase amplitude vector at the corresponding object point. The image value is mapped to the gray level interval of [0, 255] to obtain the speckle image gray level map, as shown in Figure 1C. When the scattering medium on the surface of the observed object moves, the change in light intensity leads to a change of gray distribution within the speckle image, resulting in the phenomenon of speckle particles constantly flickering. The corresponding mathematical expression of this phenomenon is that the gray value of speckle gray map fluctuates with time.

This characteristic of dynamic speckle can be used to measure particle flow imaging. The fluctuation degree of dynamic speckle reflects the velocity of scattering medium (blood flow). In the theory of dynamic speckle field, the auto-correlation function of electric field *E(t)* is used to quantify the time fluctuation of speckle. However, the fluctuation degree of electric field intensity cannot be directly measured, so the auto-correlation function of gray value *I(t)* is usually sought to derive the electric field auto-correlation function. The auto-correlation function $g\left(\tau \right)$ of gray value is expressed as:

$$g\left(\tau \right)=\frac{<I\left(t\right)I\left(t+\tau \right)>}{{<I\left(t\right)>}^{2}}$$ (1)

Where τ is decay time, a key parameter reflected $g\left(\tau \right)$ . τ is affected by the integration time (exposure time) *T* in the measurement.

In order to ensure the comparability among multiple tested objects (cases), the dimensionless average peripheral blood flow perfusion is used as a mathematical quantity to quantify the blood flow activity. Define perfusion index (*PI*) as the ratio of the exposure time *T* of the CCD and the auto-correlation decay time of speckle image fluctuation $\tau_{c}$ :

$$PI=T/\tau_{c}$$ (2)

Where *T* is the charge coupled device (CCD) exposure time representing the time range of light intensity integration. $\tau_{c}$ is the auto-correlation decay time of speckle image fluctuation, which reflects the speed of electric field intensity fluctuation, and the speed of electric field intensity fluctuation is positively correlated with the speed of scattered motion.

*K* is defined as the ratio of the standard deviation of light intensity $\sigma_{I}$ and the average value *I* represented by the gray values of all the pixels in the selection window:

$$K=\sigma_{I}/I$$ (3)

**FIGURE 2:**
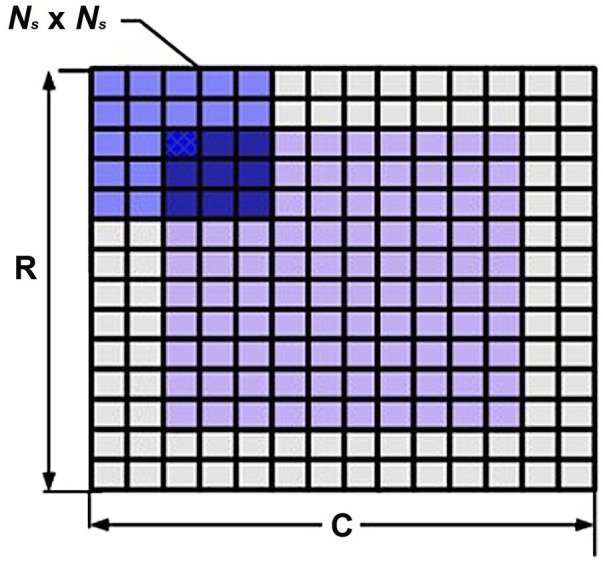
Selected window in the measurement area.

Select a window *Ns*×*Ns* in the measurement area R×C, as shown in Figure 2. $\sigma_{I}$ is calculated as the standard deviation of light intensity *I*.

The value of the contrast ratio *K* is in the range of zero-one and describes the relative speed in the original image. *K* is negatively correlated with the speed of motion of the scattering material.

The relationship between *K* and the electric field correlation function can be derived using the auto-correlation function $g\left(\tau \right)$ , light intensity auto-correlation function, and Siegert relationship between the two. This is shown in Eq. 4

$$K=\sqrt{\beta \frac{e^{-2x}+2x-1}{2x^{2}}}$$ (4)

Where $x=T/\tau_{c}=PI$ . β is a stable system parameter and is generally omitted or defaulted to 1.

The Eq. 5 can be obtained from Eqs. 3, 4:

$$\sigma_{I}/I=\sqrt{\beta \frac{e^{-2PI}+2PI-1}{2{PI}^{2}}}$$ (5)

Solve the equation to get PI of the selected window. Each selection window on the measurement area is calculated one by one to get the distribution of PI in the measurement area. The LSCI imaging is the pseudo-color of the PI distribution.

### Peripheral perfusion index estimation

Changes in peripheral perfusion reflect changes in pulsatile blood flow caused by changes in arterial and venous vascular tone (Lima and Bakker, 2014; Rasmy et al., 2015). The peripheral PI was influenced by pulse oximetry and the rate of pulsatile to non-pulsatile flow. Therefore, the PI calculation of pixels cannot reflect the distribution of PI, and the spatial or temporal statistical results of PI need to be calculated.

**FIGURE 3:**
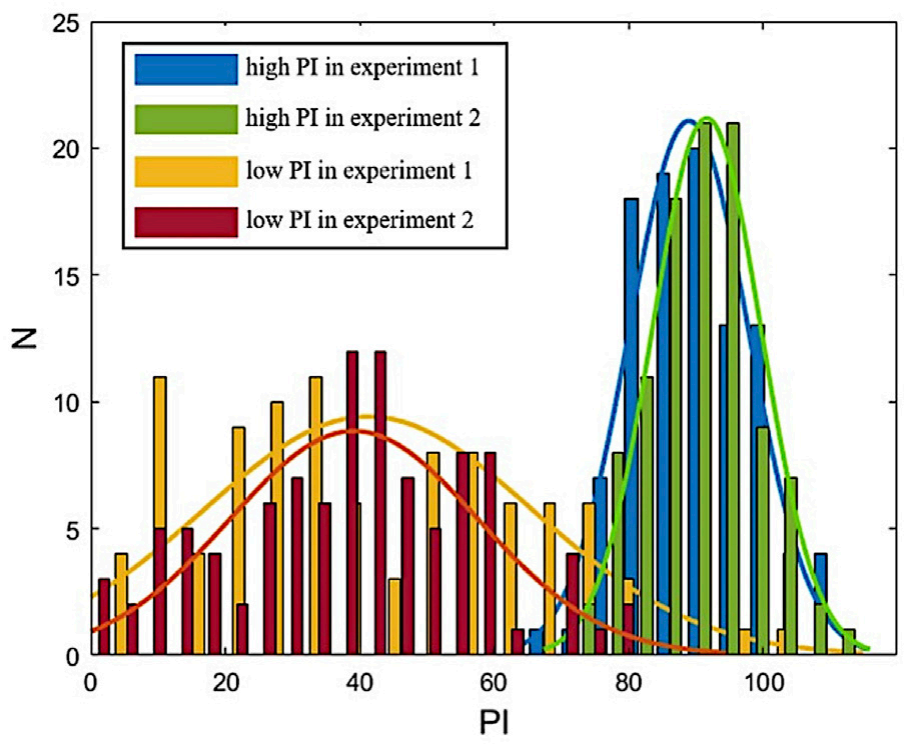
Distribution of the PI in experiments.

The implementation method of spatial PI estimation was as follows (Figure 3):

Step 1 Select the calculation window to calculate the average gray value *I* and variance of gray value *I* of pixels in the window

Step 2 Solve Eq. 4 to get the PI value of the selected window

Step 3 Move the center of the selected window to the next pixel and calculate the PI of the window

Step 4 Repeat steps 1–3 until the PI of the measured area is calculated

Step 5 If PI of the measurement area presents a normal distribution, calculate the average value of PI in the measurement area.

During the measurement, the average of PI was calculated twice. The first calculation gave the average of multiple PI in the measured area. The second calculation is the mean value of PI over time during the measurement time. The calculated results present the temporal and spatial characteristics of PI. The statistical results of repeated measurements are normally distributed, as shown in Figure 3.

### Experimental method of laser speckle contrast imaging

**FIGURE 4:**
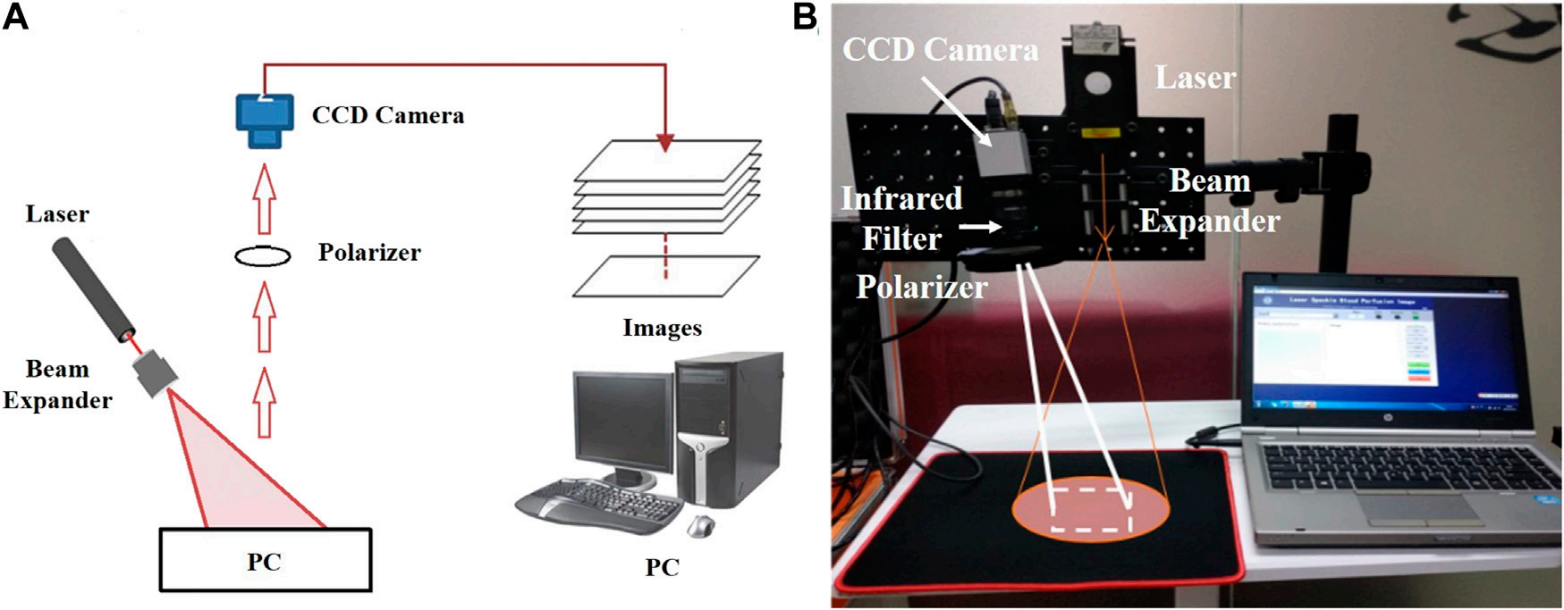
Laser Speckle Contrast Imaging set-up **(A)** Experimental schematic diagram of the speckle imaging; **(B)** Portable Experimental System.

Based on the principle of laser speckle contrast imaging, the following equipment was used to build the measuring device shown in Figure 4A: infrared laser (wavelength: 785 nm, power: 75 mW), manufacturer: Shanghai dream lasers technology; Camera Baumer txg4nir, manufacturer Baumer Electronics (Germany); Optical lens (filter, Polarizer), manufacturer Thorlabs.

In this experiment, a semiconductor laser was used to emit laser light, which was uniformly irradiated on the tested skin after passing through the beam expander. The diameter of the spot area was 10–20 cm (adjustable). The image acquisition device used a near-infrared CCD camera (exposure time: 100 ms, resolution: 1,392 × 1,040 pixels, pixel size: 6.45 μm *6.45 μm). An infrared filter and a polarizer were installed in front of the lens of the CCD camera to eliminate the interference of background stray light and shield the reflected light respectively. This process can make the imaging result clearer and more accurate. The collected image was sent to the computer and transformed into a pseudo color image with flow velocity information through algorithm processing. The patients were divided into the septic shock group and sepsis group. The laser speckle contrast imaging system (Figure 4B) was used to perform speckle contrast imaging on the finger. The continuous monitoring time was 200 s, and a pseudo color image containing flow velocity information was obtained every 5 s. As the blood circulation in the fingertip area is the most abundant, the fingertip areas of the index finger, middle finger and ring finger of each patient was used for data analysis. The principle of area selection was to include as much of the fingertip as possible but not beyond the boundary of the finger.

In this research, we choose a rectangular area of 100 × 100 pixels. The average contrast ratio of the selected area was calculated and converted into the average relative velocity value through Eqs 4, 5. The average PI was calculated as the final measurement result for statistical processing. LSCI was done using infrared laser (wavelength: 785nm, power: 75 mW), (Shanghai dream lasers technology, China); Camera Baumer txg4nir, (Baumer Electronics, Germany); Optical lens (filter, Polarizer), (Thorlabs, US). A semiconductor laser was used to emit laser light, which was uniformly irradiated on the tested skin after passing through the beam expander. The diameter of the spot area was 10–20 cm (adjustable). The image acquisition device used a near-infrared CCD camera (exposure time: 100 ms, resolution: 1,392 × 1,040 pixels, pixel size: 6.45 μm × 6.45 μm). An infrared filter and a polarizer were installed in front of the lens of the CCD camera to eliminate the interference of background stray light and shield the reflected light respectively. This process can make the imaging result clearer and more accurate. The collected image was sent to the computer and transformed into a pseudo color image with flow velocity information through algorithm processing. Patients were divided into the septic shock and sepsis groups, and a speckle contrast imaging of the finger was performed. The continuous monitoring time was 200 s, and a pseudo color image containing flow velocity information was obtained every 5 s. As the blood circulation in the fingertip area is the most abundant, the fingertip areas of the index finger, middle finger and ring finger of each patient were used for data analysis. The principle of area selection was to include as much of the fingertip as possible but not beyond the boundary of the finger. For this study, a rectangular area of 100 × 100 pixels was chosen. The average contrast ratio of the selected area was calculated and converted into the average relative velocity value through Eqs 4, 5. The average PI was calculated as the final measurement result for statistical processing.

### Statistical analyses

For continuous quantitative data, means ± standard deviation, median (interquartile range) and number (percentage) were used for normally distributed, non-normally distributed and qualitative data, respectively. Unpaired Student’s two-tailed *t*-test [Mean artery pressure (MAP), Heart-rate (HR), Body mass index (BMI), APACHEⅡ) or non-parametric Mann-Whitney U-test (Age, Sequential organ failure assessment (SOFA), Platelet distribution with (PDW), Red Blood cell distribution width (RDW), Lactic acid (Lac), CRP, Procalcitonin (PCT), White blood cell (WBC), Oxygensaturation (SpO_2_), Temperature (TM), Perfusion index (PI)] were used where appropriate. Qualitative data, such as gender, were compared using chi-square test. Receiver operator characteristic (ROC) curve analysis was performed to identify the cutoff value of PI with the best sensitivity and specificity to predict outcome in patients with sepsis and septic shock. The SOFA, APACHE Ⅱ, PDW, RDW, Lac, CRP, PCT, WBC, BMI, HR, MAP, SPO_2_, TM, and PI were assessed using a spearman correlation. R version 4.2.1 (http://www.R-project.org) and SPSS software version 22.0 (SPSS, Inc., Chicago, IL, United States) were used for statistical analysis, with *p* < .05 being statistically significant.

## Results

### Baseline data

**FIGURE 5:**
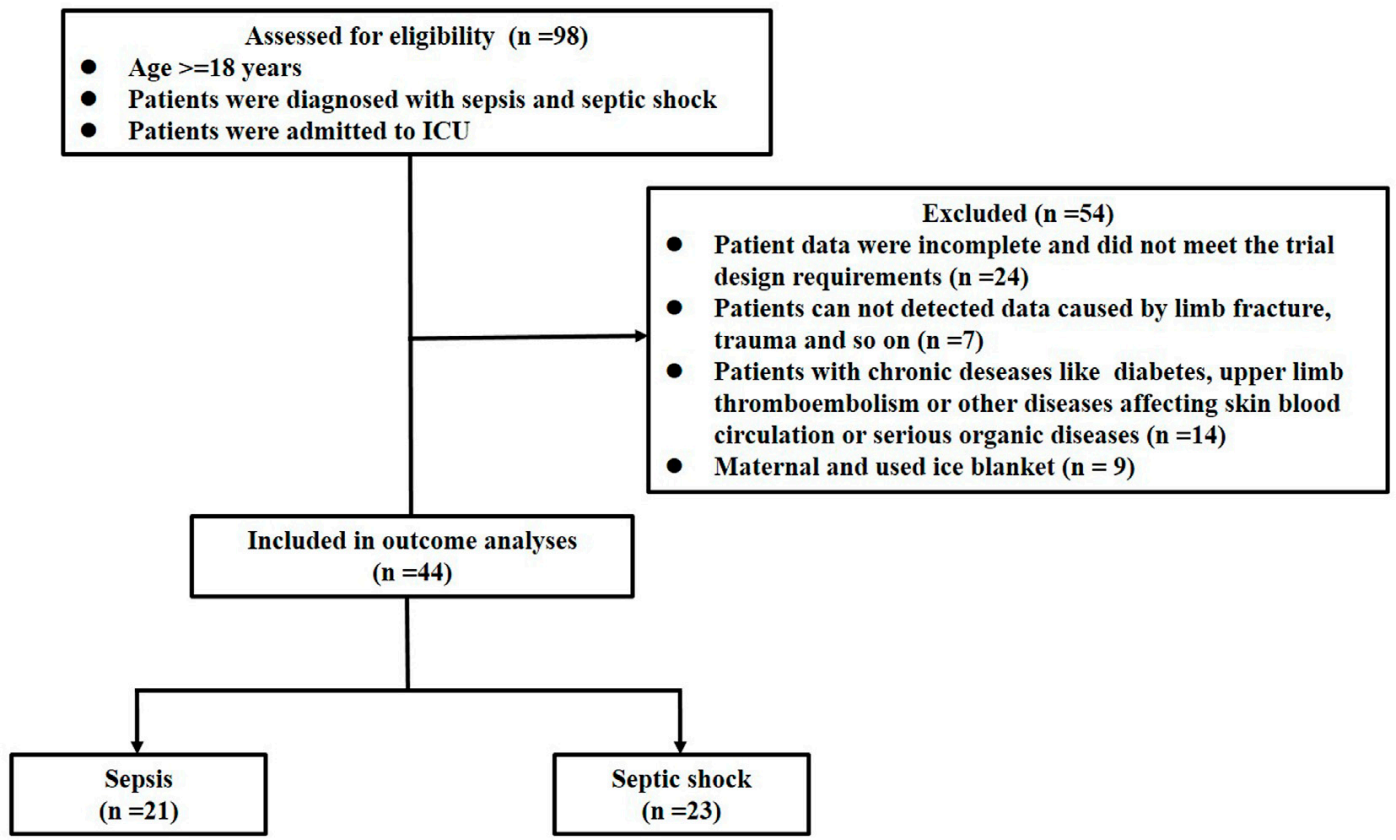
Flowchart of trial procedures.

From 1 July 2021, to 31 January 2022, 98 patients with septic shock and sepsis were diagnosed at the Department of Anesthesiology and critical care in Xinhua Hospital Affiliated with the Medical College of Shanghai Jiaotong University. Of them, 54 patients were excluded based on the exclusion criteria. Twenty-three patients comprised the septic shock group and 21 patients comprised the sepsis group (Figure 5). The median age of patients was 64 years. Both groups were similar in gender and age characteristics, and had comparable SpO_2_, TM, HR, BMI, WBC, CRP, Lac, RDW, and PDW (Table 1). However, two groups differed significantly in the mean arterial pressure, PCT, APACHE Ⅱ, SOFA and mean blood flow perfusion in the peripheral blood. Mortality rate of septic shock patients at 30 days was 39.1%, which was higher than that of septic patients (14.3%), but the difference was not statistically significant (*p* > .05).

**TABLE 1:** p-value for baseline data between the sepsis and septic shock group.

	ALL	Sepsis	Septic shock	*p*-value
	(*n* = 44)	(*n* = 21)	(*n* = 23)	
Sex (male, %)	30 (68.2)	14 (66.7)	16 (69.6)	1
Age (year, IQR)	64.0 [55.8; 69.2]	64.0 [56.0; 69.0]	61.0 [56.5; 69.5]	.556
TM (°C, IQR)	36.7 [36.5; 36.9]	36.6 [36.5; 36.8]	36.8 [36.5; 37.0]	.422
**SpO_2_ (%, IQR)**	99.5 [98.0; 100]	99.0 [98.0; 100]	100 [97.5; 100]	.472
MAP (mmHg, mean ± SD)	85.5 ± 14.8	93.3 ± 14.3	78.5 ± 11.6	.001**
HR (BPM, mean ± SD)	92.9 ± 20.8	89.2 ± 18.7	96.3 ± 22.5	.262
**BMI (kg/m^2^, mean ± SD)**	22.5 ± 4.1	22.6 ± 4.1	22.4 ± 4.2	.853
WBC (*10^9^/L, IQR)	11.5 [9.26; 17.0]	10.7 [9.58; 15.3]	12.2 [9.11; 17.8]	.217
PCT (ng/mL, IQR)	1.00 [.30; 4.34]	0.38 [.19; 1.42]	1.87 [.88; 6.16]	.003**
CRP (mg/L, IQR)	56.0 [20.0; 130]	43.0 [5.00; 129]	85.0 [31.0; 145]	.176
**Lac (mmol/L, IQR)**	2.10 [1.30; 4.00]	1.90 [1.20; 2.70]	2.40 [1.55; 9.00]	.055
RDW (IQR)	14.6 [13.3; 16.8]	13.4 [13.0; 16.5]	14.9 [14.2; 18.3]	.105
PDW (IQR)	16.5 [15.9; 17.2]	16.3 [15.9; 17.3]	16.7 [16.0; 17.0]	.647
**APACH Ⅱ(mean ± SD)**	16.9 ± 8.31	14.3 ± 7.3	19.3 ± 8.63	.047*
SOFA (IQR)	6.50 [2.00; 11.0]	2.00 [2.00; 5.00]	11.0 [7.50; 14.0]	<.001**
Source of infection (%)	**—**	**—**	**—**	.96
Lung	13 (29.5)	7 (33.3)	6 (26.1)	**—**
Abdomen	20 (45.5)	9 (42.9)	11 (47.8)	**—**
Urinary tract	2 (4.5)	1 (4.8)	1 (4.3)	**—**
Other	9 (20.5)	4 (19.0)	5 (21.7)	**—**
Mortality (death, %)	12 (27.3)	3 (14.3)	9 (39.1)	.131
PI (IQR)	26.3 [18.5; 33.9]	35.3 [31.9; 43.7]	18.6 [16.1; 22.2]	<.001**

*p < .05 **p < .01 Abbreviations: MAP, Mean artery pressure; HR, Heartrate; BMI, Body mass index; APACHEⅡ, acute physiology and chronic health evaluation; SOFA, Age, Sequential organ failure assessment; PDW, Platelet distribution with; RDW, Red Blood cell distribution width; Lac, Lactic acid; CRP, C-reactive protein; PCT, Procalcitonin; WBC, White blood cell; SpO_2_, Oxygensaturation; TM, Temperature; PI, Perfusion index. Outcome: 30-day mortality.

### LSCI measurements

**FIGURE 6:**
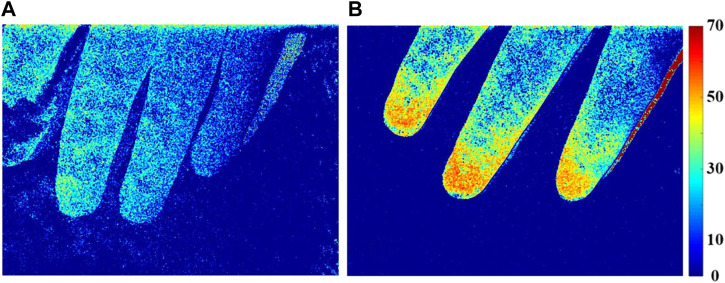
Perfusion index (PI) distribution of finger microcirculation blood flow in patients with septic shock **(A)** and sepsis **(B)**.

We next performed laser speckle contrast imaging and measurements for both patient groups. The median PI was significantly lower in the septic shock patients than in sepsis patients, 35.3 vs. 18.6, respectively (Table 1 *p* < .01). Microcirculation in sepsis and septic shock patients is further demonstrated by pseudo color images (Figure 6).

The microcirculatory blood flow of patients with sepsis (Figure 6B) was more active than that of patients with septic shock (Figure 6A), and the characteristic area of blood flow perfusion was mainly concentrated in the fingertip area.

**FIGURE 7:**
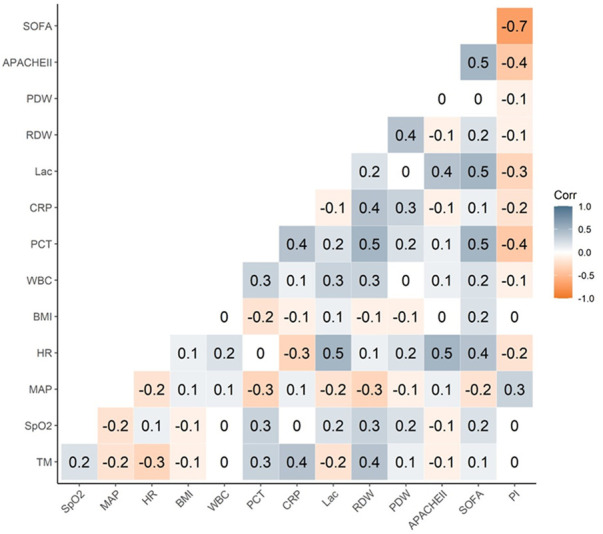
The R value of relevance.

PI negatively correlated with APACHE Ⅱ, *p* = .01 < .05 and r = −.4, SOFA score, *p* < .001 and r = −.7, and lactate level, *p* = .01 < .05 and r = −.3, and was related to the severity of the disease and overall sepsis in the peripheral microcirculation (Table 2; Figure 7).

**TABLE 2:** *p*-value for the relevance.

*p*-value	TM	SpO_2_	MAP	HR	BMI	WBC	PCT	CRP	Lac	RDW	PDW	APACHE Ⅱ	SOFA	PI
TM	0	—	—	—	—	—	—	—	—	—	—	—	—	—
SpO_2_	.39	0	—	—	—	—	—	—	—	—	—	—	—	—
MAP	.46	.35	0	—	—	—	—	—	—	—	—	—	—	—
HR	.09	.44	.25	0	—	—	—	—	—	—	—	—	—	—
BMI	.69	.61	.2	.58	0	—	—	—	—	—	—	—	—	—
WBC	.75	.89	.99	.05	.59	0	—	—	—	—	—	—	—	—
PCT	.53	.39	.3	.54	.75	.12	0	—	—	—	—	—	—	—
CRP	.02*	.72	.82	.05	.98	.99	.07	0	—	—	—	—	—	—
Lac	.17	.41	.06	0**	.73	.06	.54	.08	0	—	—	—	—	—
RDW	.47	.1	.53	.88	.56	.35	.11	.01*	.73	0	—	—	—	—
PDW	.83	.71	.49	.3	.8	.75	.94	.12	.1	.05	0	—	—	—
APACH Ⅱ	.27	.44	.92	0**	.92	.21	.92	.43	0**	.12	.74	0	—	—
SOFA	.5	.24	.04*	.02*	.25	.12	.02*	.77	0^**^	1	.7	0**	0	—
PI	.98	.8	.14	.36	.97	.54	.2	.22	.01*	.46	.77	.01*	0**	0

*p < .05, **p < .01.

There was no significant correlation between the PI of peripheral microcirculation and the TM, SpO_2_, MAP, HR, BMI, RDW, PDW, CRP, and PCT (Table 2; Figure 7).

We found that the average peripheral blood flow PI of septic shock patients was significantly lower than that of septic patients, with the median cutoff value of 26.25.

**FIGURE 8:**
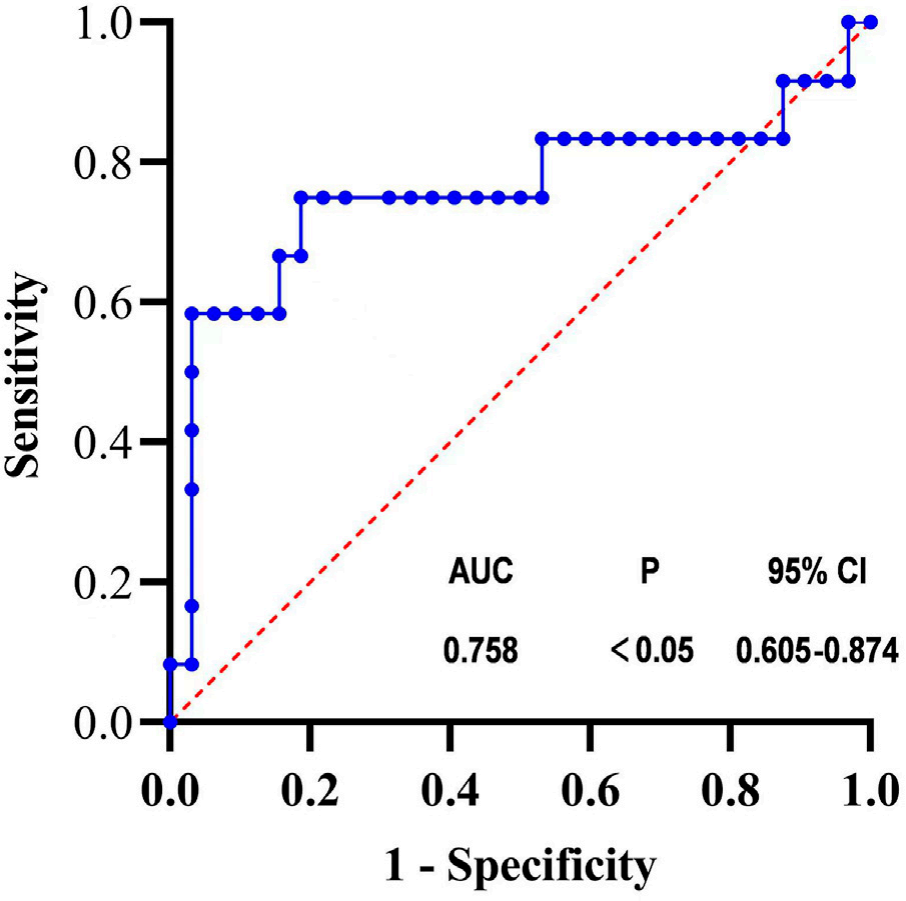
ROC curve of PI.

PI significantly correlated with the group sepsis and septic shock (*p* < .001, r = −.865). And PI significantly correlated with the outcome or mortality (*p* = .007 < .05, r = −.398). Next, we divided the group with outcome and see the Supplementary Table S1. ROC curve was calculated for PI and the sensitivity was 81.3%, the specificity was 75% when PI cutoff value chooses 20.88 and see Figure 8.

## Discussion

Laser speckle technology was used in this study to monitor the microcirculation of patients with septic shock and provide more precise monitoring indicators for their treatment. Our results show that the microcirculation of patients with septic shock is significantly different than patients with sepsis, and really highlights the importance of monitoring the microcirculation in these patients. Laser speckle contrast imaging is a simple system structure, with fast imaging speed and flexible algorithm. The combination of laser and CCD camera allows visualizing large areas using high-resolution rapid blood flow imaging. The algorithm not only corrects the artifacts caused by muscle shaking due to uneven breathing, variable heart rate and drug stimulation, but also improves the signal-to-noise ratio of blood flow distribution.

Sepsis is a life-threatening organic dysfunction caused by the uncontrolled response of the host to infection. The resulting septic shock is generally caused by acute reduction of effective circulating blood volume, decrease in the extent of microcirculation perfusion, changes in the cellular metabolism and damage of main organs caused by various pathogenic factors (Rhodes et al., 2017), and is associated with high mortality rate (38.5%). Septic patients exhibit heterogeneous blood flow patterns of microcirculation, which, in turn, result in hypoperfusion of tissues and reduced oxygen extraction and usage abilities of the cells, and impaired organ function (Rivers et al., 2001). Therefore, adequate maintenance of perfusion and organ homeostasis remain important therapeutic targets. Although systemic perfusion is traditionally managed by microcirculation monitoring, recent studies showed that monitoring of peripheral microcirculation, especially in non-vital organs, can predict survival and help in better understanding the dynamics of organ failure (Vellinga et al., 2015; Orbegozo et al., 2018).

Currently, there are various methods for monitoring the microcirculation (Ocak et al., 2016), using Side-stream Dark Field (SDF) technology as the most widely used. This technique visualizes the microcirculation more clearly and directly but can be affected by the representativeness of local tissue mucosa and the complexity of volume judgment. The microcirculation of sublingual or oral mucosa can be directly or indirectly assessed at the bedside through orthogonal polarization spectral imaging (OPS), dark field image analysis (lateral flow dark field SDF) or laser Doppler blood flow measurement (laser Doppler blood flow measurement LDF). These technologies adopt a semi quantitative method, and the results can be affected by the human factor. Moreover, there is a possibility of bubbles on the surface of the monitoring object that may impact the imaging, and it is difficult to monitor the sublingual mucosa in patients with endotracheal intubation (Tang and Wang, 2011). At the same time, our previous research also found the shortcomings of near-infrared technology in monitoring the oxygen saturation of the center. When the tissue perfusion is insufficient, the monitoring value of near-infrared technology differs greatly from the actual value (Ruan et al., 2015). In addition, while SDF is currently used to measure sublingual microcirculation, many patients with septic shock cannot complete the detection due to various reasons. Therefore, we introduced laser speckle technology to further monitor the microcirculation of patients with septic shock and provide more precise monitoring indicators for their treatment. In 2014, the European guideline for monitoring circulatory shock and hemodynamics and the consensus of Chinese emergency clinical practice experts on acute circulatory failure successively stated that hemodynamic treatment should be guided by tissue perfusion, and tissue perfusion should be guaranteed during the initial period of shock admission. Even when the hemodynamic parameters of patients with septic shock are stable, tissue perfusion should still be monitored to protect organ function (Cecconi et al., 2014; Emergency Medical Branch of Chinese Medical Doctor Association, 2016). The results of the current study clearly show the differences in the microcirculation of patients with septic shock and with sepsis.

Our results show that the use of laser speckle contrast imaging technology with pseudo color images, allows clinicians greater capacity to observe the changes of finger-tip microcirculation in patients with septic shock, thus providing detection means and indicators for more effective clinical treatment. Many previous studies have pointed out that during septic shock, the effective circulating blood volume of the whole body is significantly reduced, and that the whole-body hypoxia causes blood redistribution. To ensure the perfusion of the heart and brain and other important organs, the blood vessels of the mesentery and extremities contract, and the blood flow of the intestine and extremities is significantly reduced. While many studies have selected the mesentery blood flow (Ding et al., 2014) to monitor perfusion, these measurements are not easy to obtain. Therefore, this study uses the fingertip measurements, with the help of laser speckle technology.

We analyzed the previously reported indicators that may be related to sepsis and microcirculation (Boillot et al., 2013; Cecconi et al., 2014; Puskarich et al., 2016; Fontana et al., 2017; Hohenauer et al., 2019; Rubins et al., 2019; Mok et al., 2021). Our study found that the average peripheral blood perfusion of the fingertip negatively correlated with the SOFA score, which was consistent with previous studies (Puskarich et al., 2016). SOFA score evaluates the extent of sepsis on various organs and reflects the severity of the disease.

We found that PI also correlated with the levels of lactate, which was different from previous studies that showed no association between the extent of lactate clearance and changes in microcirculatory blood flow in patients with septic shock (Puskarich et al., 2016). Although some studies have found (Puskarich et al., 2016) that microcirculation is not correlated with APACHE Ⅱ score, our study found that PI is negatively correlated with APACHE Ⅱ, which is controversial.

In addition, we also found relatively no correlation between PI and body temperature, oxygen saturation, average arterial pressure, heart rate, BMI, white blood cells, average distribution width of red blood cells, average width of platelets, CRP and PCT, which is also consistent with some studies (Boillot et al., 2013; Fontana et al., 2017; Rubins et al., 2019; Mok et al., 2021). MAP differed significantly between the sepsis and the septic shock groups. However, we did not find a correlation between MAP and PI. This may be related to the vasoactive drugs used by the patients. Although different vascular drugs have no significant differences in their effect on microcirculation in septic shock patients, they may affect microcirculation in patients with sepsis and septic shock in a different way (Cecconi et al., 2014). We did not analyze the effect of different vasoactive drugs on PI, due to the small number of patients taking the medications. PI was related to the patient mortality and showed a negative correlation (r = −.398). ROC curve was calculated for PI and the sensitivity was 81.3%, the specificity was 75% when PI cutoff value chooses 20.88. Mottling is patchy skin discoloration that reflects blood flow reduction and skin hypoperfusion. Furthermore, the mottling score at 6 h has been shown to be associated with increased mortality (J Intensive Care Med, 2021 December; 36 (12):1,385–1,391). All this proved that microcirculatory was associated with mortality. As the same results, we found that the average peripheral blood perfusion was closely related to the outcome of patients.

Some studies found (Hohenauer et al., 2019) that the peripheral temperature can affect the peripheral microcirculation. We did not find the correlation between the body temperature and the relative velocity of the peripheral blood flow. To avoid the influence of the body temperature, we excluded patients with high body temperatures and patients that required the ice blanket machine.

LSCI technology has certain limitations. Due to the insufficient penetration of near-infrared light, it can only be used for blood flow imaging of the epidermis and cannot be used for blood flow imaging of deep tissues. As mentioned above, like with other blood flow imaging techniques, only PI can be measured by LSCI. With further improvement of hardware and software algorithms, the measurement accuracy can be improved while ensuring the accuracy of the measurement results.

Our study has certain limitations. This study only included patients who were diagnosed with septic shock 1 h after the admission. Some patients had been in shock before ICU admission, and therefore, the hemodynamics was not accounted for. This may have caused a certain bias in the study. Additionally, we did not continuously monitor patients’ indices in this study, as it is difficult to achieve in sepsis and septic shock patients due to the variability on the length of needed observation and a need in constant presence of personnel at the bedside. Further studies in animal models with continuous observation are needed. The present study is limited by the sample size which impacted statistical significance of some of the results. That may explain the lack of difference in the mortality rates in patients with sepsis and with septic shock. However, despite lack of difference in mortality, we observed a difference in PI even with the small sample size of this study. As a next step, we may further increase the sample size and study whether PI has a guiding effect on fluid resuscitation, etc. Some studies have found that sedatives have an effect on microcirculation (Brookes et al., 2004; Klein et al., 2011), and this effect was not considered in our study.

## Conclusion

The laser speckle contrast imaging method can efficiently detect the finger-tip microcirculation in septic shock patients, and is reliable for both sepsis and septic shock patients. The results showed that PI negatively correlated with APACHE Ⅱ, SOFA score, lactate and mortality, which provides useful and reliable auxiliary information for the diagnosis and monitoring of septic shock.

## Data Availability

The original contributions presented in the study are included in the article/Supplementary Material, further inquiries can be directed to the corresponding authors.

## Abbreviations

LSCI, Laser Speckle Contrast Imaging; PI, perfusion index; APACHE, Acute physiology and chronic health evaluation; SOFA, Sequential organ failure assessment; CRP, C-reactive protein; SDF, side-stream dark field; MAP, Mean artery pressure; HR, Heart rate; BMI, Body mass index; PDW, Platelet distribution width; RDW, Red blood cell distribution width; PCT, Procalcitonin; Lac, lactic acid; WBC, White blood cell; SpO2, Oxygen saturation; TM, temperature; OPS, orthogonal polarization spectral imaging.

## Supplementary Material

SUPPLEMENTARY TABLE S1 The *p*-value of baseline data.
