# Supplementary material for: Laser speckle contrast imaging to monitor microcirculation: An effective method to predict outcome in patients with sepsis and septic shock
Source: Front Bioeng Biotechnol. 2023 Jan 11;10:1067739. doi: 10.3389/fbioe.2022.1067739 (PMC9890168; doi:10.3389/fbioe.2022.1067739)
Supplement: Supplementary file 1 [file Table1.docx]

Supplementary table 1 P value for baseline data of the outcome

|  | [ALL] N=44 | discharge N=32 | death N=12 | P value |
| --- | --- | --- | --- | --- |
| Sex: |  |  |  | 0.722 |
| female | 14 (31.8%) | 11 (34.4%) | 3 (25.0%) |  |
| male | 30 (68.2%) | 21 (65.6%) | 9 (75.0%) |  |
| Age | 64.0 [55.8;69.2] | 61.5 [53.5;66.8] | 66.0 [63.0;71.8] | 0.048* |
| TM | 36.7 [36.5;36.9] | 36.7 [36.5;36.9] | 36.5 [36.2;36.9] | 0.112 |
| SpO_2_ | 99.5 [98.0;100] | 99.0 [97.0;100] | 100 [99.8;100] | 0.045 |
| MAP | 85.5 (14.8) | 87.2 (14.4) | 81.2 (15.6) | 0.263 |
| HR | 92.9 (20.8) | 87.3 (17.9) | 108 (21.3) | 0.008** |
| BMI | 22.5 (4.08) | 22.8 (4.15) | 21.5 (3.89) | 0.327 |
| WBC | 11.5 [9.26;17.0] | 11.0 [8.21;16.6] | 12.1 [10.9;20.2] | 0.225 |
| PCT | 1.00 [0.30;4.34] | 0.78 [0.20;2.53] | 1.85 [1.23;4.39] | 0.108 |
| CRP | 56.0 [20.0;130] | 50.5 [16.0;160] | 62.0 [35.8;87.2] | 0.607 |
| Lac | 2.10 [1.30;4.00] | 1.85 [1.28;3.10] | 6.50 [2.18;10.4] | 0.016* |
| RDW | 14.6 [13.3;16.8] | 14.1 [13.3;16.6] | 15.2 [14.8;17.8] | 0.123 |
| PDW | 16.5 [15.9;17.2] | 16.4 [15.9;17.2] | 16.6 [16.2;17.0] | 0.626 |
| APACHEII | 16.9 (8.31) | 15.7 (7.52) | 20.2 (9.73) | 0.169 |
| SOFA | 6.50 [2.00;11.0] | 5.00 [2.00;8.25] | 12.0 [8.25;14.0] | 0.009** |
| Shock: |  |  |  | 0.131 |
| NO | 21 (47.7%) | 18 (56.2%) | 3 (25.0%) |  |
| YES | 23 (52.3%) | 14 (43.8%) | 9 (75.0%) |  |
| PI | 26.3 [18.5;33.9] | 27.9 [22.5;35.6] | 16.4 [15.1;23.0] | 0.009** |
| Infection: |  |  |  | 0.554 |
| Lung | 13 (29.5%) | 9 (28.1%) | 4 (33.3%) |  |
| Abdomen | 20 (45.5%) | 13 (40.6%) | 7 (58.3%) |  |
| Urinary_tract | 2 (4.55%) | 2 (6.25%) | 0 (0.00%) |  |
| other | 9 (20.5%) | 8 (25.0%) | 1 (8.33%) |  |

* P<0.05 ** P<0.01 Abbreviations: Mean artery pressure (MAP), Heartrate (HR), Body mass index (BMI), acute physiology and chronic health evaluation (APACHEⅡ), Age, Sequential organ failure assessment (SOFA), Platelet distribution with (PDW), Red Blood cell distribution width (RDW), Lactic acid (Lac), C-reactive protein (CRP), Procalcitonin (PCT), White blood cell (WBC), Oxygensaturation (SpO_2_), Temperature (TM), Perfusion index (PI). Outcome: 30-day mortality
